# Association de neuroleptiques atypiques avec les anticonvulsivants et syndrome malin (à propos de deux cas)

**DOI:** 10.11604/pamj.2014.18.220.4868

**Published:** 2014-07-16

**Authors:** Fadoua Oueriagli Nabih, Abdeslam Benali, Imane adali, Fatiha Manoudi, Fatima Asri

**Affiliations:** 1 Service de Psychiatrie, Hôpital Militaire Avicenne, Equipe de Recherche pour la Santé Mentale, Université Caddi Ayyad, Marrakech, Maroc

**Keywords:** Amisulpride, carbamazepine, syndrome malin, olanzapine-valproate de sodium, Amisulpride, carbamazepine, malignant syndrome, olanzapine sodium valproate

## Abstract

Le syndrome malin des neuroleptiques (SMN) est une complication rare mais grave du traitement par les neuroleptiques, pouvant engager le pronostic vital. Les auteurs rapportent deux observations, la première d'une jeune patiente de 18 ans, suivie pour une épilepsie partielle temporale, sous carbamazépine (800mg/jour) depuis 13 ans, et qui a développé un SMN après introduction d'amisulpride (600 mg/jour). La deuxième observation d'un jeune patient de 28 ans sous valproate sodium (750mg/jour) depuis 10 ans et qui a présenté un SMN après association d'olanzapine (20mg/jour). Les cliniciens doivent être vigilants par rapport au risque d'induction d'un SMN après introduction de neuroleptiques atypiques chez des patients traités pendant une longue durée avec des anticonvulsivants.

## Introduction

Le syndrome malin des neuroleptiques (SMN), décrit en 1960 par Delay et al. [1] sous le nom de syndrome akinétique hypertonique, représente un accident rare, mais redoutable pouvant engager le pronostic vital. Si la relation du SMN avec les neuroleptiques classiques est une donnée acquise, plusieurs auteurs ont aussi rapporté des cas de SMN faisant suite à l'association de neuroleptiques atypiques et d'autres médicaments tels les anticonvulsivants. Nous en rapportons deux cas de SMN suite à l'association de neuroleptique atypique et d'anticonvulsivant.

## Patient et observation

### Première observation

Il s'agissait d'une jeune patiente de 18 ans, suivie pour une épilepsie partielle temporale, sous carbamazépine 800 mg / j depuis l’âge de 04 ans. Elle était admise au service de psychiatrie pour troubles de comportements, la patiente avait présenté une instabilité psychomotrice, un délire de persécution et mystico-religieux et des hallucinations auditives, sans troubles de la conscience. Elle avait été mise sous un neuroleptique atypique (Amisulpride 600mg /j) et un anxiolytique benzodiazépinique (temesta 7,5mg/j). 72 heures après, la patiente avait développé une symptomatologie faite d'une obnubilation de la conscience, une hypersudation, une hypertonie plastique surtout aux membres supérieurs, un tremblement des extrémités et une hypersudation. Il n'y avait pas de déficit moteur et la motricité oculaire était conservée. Sur le plan général, elle était tachycarde, la tension artérielle était à 90/50 mm Hg. La température était à 37°C. Devant ce tableau clinique, nous avions évoqué le diagnostic de syndrome malin des neuroleptiques même en absence de fièvre. Le traitement neuroleptique fut arrêté et La malade avait été adressée au service de réanimation en urgence. La NFS, La VS et l'ionogramme étaient normaux. La créatine phosphokinase (CPK) était très élevée 2389 mu/ l. L’électroencéphalogramme (EEG) avait montré un tracé normal. L'IRM encéphalique avec protocole d’épilepsie était normale. La malade avait été réhydratée par voie parentérale et mise sous bromocriptine (à doses progressives jusqu’à 20mg/j), associé à un anxiolytique benzodiazépinique. L’évolution était favorable, avec une régression de l'hypertonie au bout d'une semaine et normalisation des CPK au bout de 03 semaines. La symptomatologie psychiatrique avait disparu progressivement sous sismothérapie.

### Deuxième observation

Patient de 28 ans, suivi pour un trouble bipolaire, sous valproate de sodium à raison de 750 mg/jour depuis 10 ans, qui a été hospitalisé dans le service de psychiatrie pour un troisième accès maniaque. Une étiologie organique du syndrome maniaque a été éliminée d'emblée: la NFS et l'ionogramme sanguin complet étaient normaux, la recherche des toxiques dans les urines était négative et l'IRM cérébrale était sans particularité. Le patient a été mis sous olanzapine à raison de 20 mg/jour et la posologie du valproate de sodium à été augmenté à 2g/jour progressivement. Un mois après le début du traitement, le patient a développé un tableau fait d'une rigidité musculaire, un tremblement de repos des extrémités, une hypersudation, des troubles de la déglutition et des troubles de la conscience. Il n'avait pas de déficit moteur et la motricité oculaire était conservée. Sur le plan général, la tension artérielle était à 103/68 mm Hg, la fréquence cardiaque à 140/min et la température à 39°C. Le traitement neuroleptique fut arrêté et Le malade avait été adressé au service de réanimation en urgence Aucun foyer infectieux n'a été retrouvé à l'examen clinique. Le bilan biologique réalisé a montré une augmentation de l'activité créatine kinase (CK) plasmatique: 1 289 U/l, la concentration plasmatique de la CRP était normale, l'examen cytobactériologique des urines (ECBU) et les hémocultures se sont avères négatifs. L’étude du liquide céphalorachidien était normale. La radiographie thoracique et L'IRM cérébrale étaient sans particularités. Le diagnostic d'un SMN a été posé après avoir éliminé une étiologie infectieuse, métabolique ou neurologique. Après réhydratation parentérale, traitement antipyrétique et traitement anti dopaminergique (bromocriptine à doses progressives jusqu’à 20mg/j), l’évolution était favorable au bout d'une semaine.

## Discussion

Le syndrome malin des neuroleptiques (SMN) est une complication rare du traitement par les neuroleptiques avec une incidence estimée entre 0,02 et 3,23% [1]. Le SMN est dû à un blocage du système dopaminergique. IL se constitue en 1 à 3 jours et la symptomatologie évolue en deux phases associant des signes de dérèglement neurovégétatif central à des signes d'imprégnation neuroleptique. Le diagnostic du SMN est parfois difficile en raison des variations cliniques et de l'absence de marqueurs biologiques spécifiques. Selon les critères DSM V, le diagnostic du SMN est retenu chez un patient après introduction d'un traitement dopaminergique si présence des critères suivants: Une élévation de la température >38°C; Une rigidité musculaire sévère; Des troubles de la conscience allant de la confusion au coma; Signes para cliniques d'atteinte musculaire: une élévation de l'activité créatine kinase plasmatique (CK) supérieur à 04 fois la normale; Troubles du système nerveux sympathiques avec présence d'au moins deux signes: (Tension artérielle labile (changement de la diastolique ≥20 mmHg ou changement de la systolique ≥25 mmHg pendant <24 heures); Tension artérielle élevée; Dysphagie; Incontinence urinaire.); Hyper métabolisme (tachypnée ou tachycardie); Absence de causes infectieuses, toxiques, métaboliques et neurologique.

Dans notre observation, nous avons retenu le diagnostic de SMN devant l'association d'une rigidité extra pyramidale, des signes d'un dérèglement neurovégétatif, des troubles de la conscience et le taux élevé des enzymes musculaires (CK) même en l'absence de fièvre. En effet, à côté des tableaux cliniques classiques, des tableaux incomplets ont été décrits. Thase et Shostak [2] avaient rapporté l'observation d'un patient présentant un tableau de rhabdomyolyse avec un taux sanguin de CK élevé et une hypertonie extrapyramidale sans fièvre. Pope et al [3], considéraient dans leur travail l'hyperthermie comme un critère diagnostique majeur du SMN, mais 11 de leurs patients avaient une température inférieure à 38°C. Angelopoulos et al [4] avait rapporté un cas de SMN sans fiévre secondaire à l'association d'amisulpride et d'oxcarbazepine.

Le SMN peut survenir à tout âge et toucher les deux sexes avec une légère prédominance masculine. Toutes les catégories de neuroleptiques sont concernées: incisifs, doux, retards et même atypiques. En effet, plusieurs cas de SMN ont été décrits dans la littérature en rapport avec des neuroleptiques atypiques tel la risperidone, la clozapine, l'olanzapine et la quetiapine^5^. Certains facteurs prédisposent plus à cette complication comme les antécédents neurologiques (confusion, agitation, épilepsie’) et les troubles hémodynamiques (déshydratation ou hyponatrémie) [6]. Certaines interactions médicamenteuses peuvent augmenter le risque de survenue d'un SMN, c'est le cas de l'association d'anticonvulsivants et de neuroleptiques atypiques [7]. Plusieurs cas ont été décrits dans la littérature, c'est le cas de Nisijima et al. [8] qui rapportent l'observation d'un patient traité par amisulpride pendant 30 ans et qui a développé un SMN après introduction de la carbamazépine à raison de 400 mg/jour. Angelopoulos et al [4] décrivent un cas de SMN après association d'oxcarbazepine et de neuroleptiques. Filice Et al. [9] rapportent le cas d'un patient qui a développé un SMN après introduction d'olanzapine (20 mg/jour) et de divalproate de sodium (500mg/jour). Dans la première observation, on peut suggérer que le jeune âge, les antécédents d’épilepsie et l'association d'amisulpride et de la carbamazépine sont des facteurs de risque qui ont favorisé la survenue du SMN. Pour la deuxième observation, l'introduction de l'olanzapine chez un patient traité pendant longtemps par du valproate de sodium a augmenté le risque de survenue du SMN.

Concernant la prise en charge, la première mesure à prendre est l'arrêt du traitement neuroleptique. Le traitement symptomatique repose sur la réhydratation par voie parentérale, l'abaissement de la température centrale, la préservation de la fonction rénale, l'assistance cardiorespiratoire et la prévention des complications de décubitus [10]. Le traitement spécifique est basé sur la bromocriptine, agoniste dopaminergique, administrée par voie orale et à doses fractionnées (7,5 à 60mg/j). Des substances myorelaxantes telles que le dantroène ou les benzodiazépines sont utilisées. Le SMN reste une complication mortelle dans 5 à 25% des cas, une évolution favorable peut se voir si le patient est pris en charge le plutôt possible.

## Conclusion

Le SMN est une complication rare mais grave du traitement par les neuroleptiques. C'est une urgence diagnostique et thérapeutique. Des associations médicamenteuses peuvent augmenter le risque de survenue du SMN, c'est le cas de l'association de neuroleptiques atypiques et des anticonvulsivants.
